# Supplementary material for: Insights into the Metabolite Differentiation Mechanism Between Chinese Dry-Cured Fatty Ham and Lean Ham Through UPLC-MS/MS-Based Untargeted Metabolomics
Source: Foods. 2025 Feb 5;14(3):505. doi: 10.3390/foods14030505 (PMC11816373; doi:10.3390/foods14030505)
Supplement: Supplementary file 1 [file foods-14-00505-s001.zip › foods-3394212-supplementary.pdf]

(a)

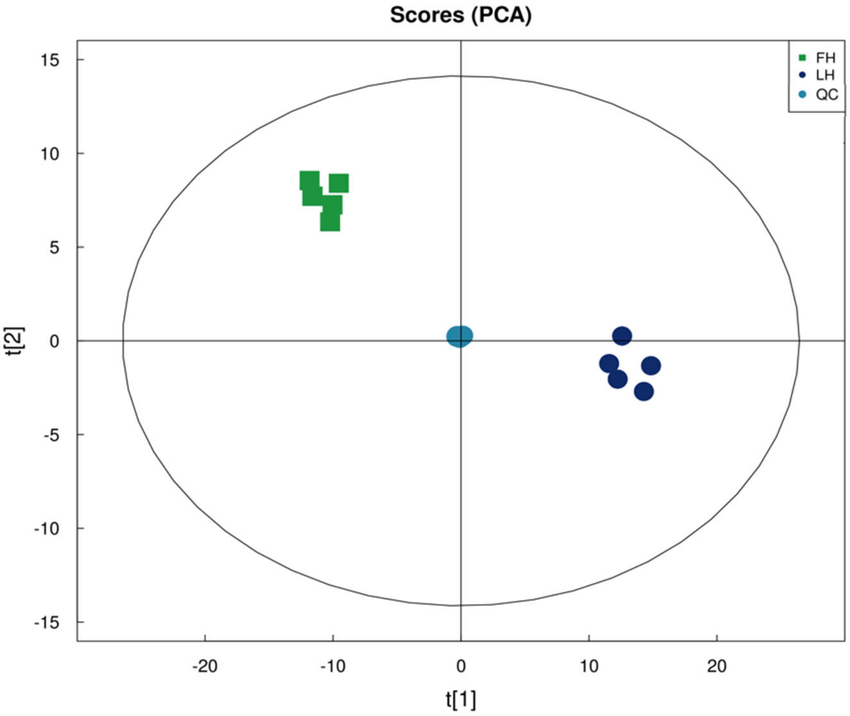

(b)

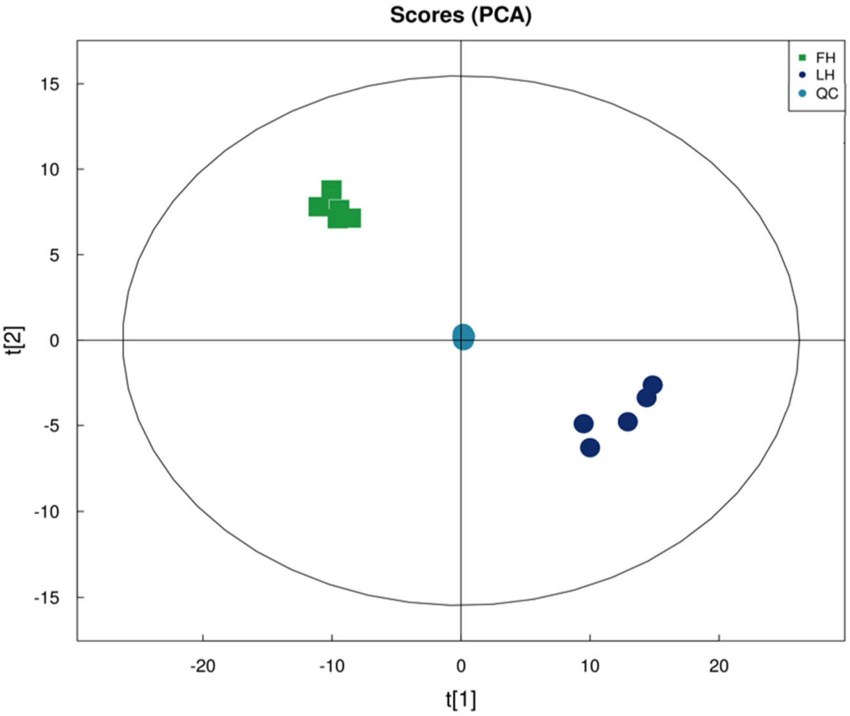

Figure S1. Principal component analysis (PCA) of metabolites from quality control (QC) and test samples at both ESI+ (a) and ESI− (b). FH, fatty ham; LH, lean ham.

Table S1 Identification results of defined metabolites from FH and LH through UPLC-MS/MS at both ESI+ and ESI−

ID	Adduct	Name	m/z	Retention time
**ESI+**				
M118T267_2	(M+H)+	Betaine	118.09	267.17
M759T43	(M+Na)+	Thioetheramide-PC	758.57	42.72
M123T62_2	(M+H)+	Nicotinamide	123.05	61.62
M258T380	M+	Glycerophosphocholine	258.11	380.46
M116T311	(M+H)+	D-Proline	116.07	311.48
M300T37	(M+H)+	Palmitoyl ethanolamide	300.29	36.55
M170T104_2	(M+H)+	Pyridoxine	170.08	103.69
M300T83	(M+H)+	Sphingosine	300.29	83.14
M227T578	(M+H)+	L-Carnosine	227.11	577.60
M277T50	(M+Na)+	cis-9-Palmitoleic acid	277.21	50.06
M162T358_3	(M+H)+	L-Carnitine	162.11	357.94
M136T157_2	(M+H)+	Adenine	136.06	157.25
M269T224	(M+H)+	Allopurinol riboside	269.09	223.55
M246T430	(M+H)+	Arg-Ala	246.15	430.42
M324T36	(M+H)+	Linoleoyl ethanolamide	324.29	35.79
M263T235	(M+H)+	Pro-Phe	263.14	234.67
M769T136	(M+H-H_2O)+	1,2-dioleoyl-sn-glycero-3-phosphatidylcholine	768.59	136.05
M233T248	(M+H)+	Thr-Leu	233.15	247.80
M205T254	(M+H)+	L-Tryptophan	205.10	253.71
M219T267	(M+H)+	Val-Thr	219.13	266.63
M272T455	(M+H)+	Pro-Arg	272.17	454.80
M124T223_2	(M+H)+	Nicotinate	124.04	222.67
M247T381	(M+H)+	Val-Glu	247.13	380.58
M269T293	(M+H)+	His-Ile	269.16	292.82

M522T186_2	(M+H)+	1-Oleoyl-sn-glycero-3-phosphocholine	522.35	186.03
M771T100	(M+H-H_2O)+	1-Stearoyl-2-oleoyl-sn-glycerol 3-phosphocholine (SOPC)	770.60	99.62
M175T374_2	(M+H)+	DL-Arginine	175.12	374.25
M231T216	(M+H)+	Val-Ile	231.17	215.68
M269T254	(M+H)+	Inosine	269.09	254.07
M277T36	(M+H)+	Stearidonic Acid	277.21	36.30
M165T296_2	(M+H)+	trans-2-Hydroxycinnamic acid	165.05	295.59
M245T202	(M+H)+	Ile-Leu	245.19	202.25
M279T188	(M+H)+	Phe-Ile	279.17	188.04
M265T204	(M+H)+	Val-Phe	265.15	204.20
M288T357	(M+H)+	Arg-Ile	288.20	356.66
M237T156	(M+H-H_2O)+	His-Val	237.13	156.05
M227T325	(2M+K)+	Dimethyl sulfone	226.99	324.88
M213T106	(M+CH_3COO+2H)+	Perillyl alcohol	213.15	105.88
M245T418	(M+H)+	Pro-Glu	245.11	417.88
M90T357	(M+H)+	L-Alanine	90.05	357.29
M218T436	(M+H)+	Ala-Lys	218.15	436.05
M338T34_2	(M+H)+	Erucamide	338.34	33.81
M219T415	(M+H)+	Ala-Glu	219.10	414.90
M160T379_2	(M+CH_3COO+2H)+	Cyclohexylamine	160.13	378.63
M274T357	(M+H)+	Val-Arg	274.19	357.04
M232T312	(M+H)+	Val-Asn	232.13	311.84
M795T134	(M+CH_3CN+Na)+	Sphingomyelin (d18:1/18:0)	794.60	134.28
M274T389_2	(M+H)+	Arg-Val	274.19	389.22
M276T458	(M+H)+	Gamma.-L-Glu-.epsilon.-L-Lys	276.15	457.96
M176T390_2	(M+H)+	L-Citrulline	176.10	390.15
M267T226	(M+H)+	Thr-Phe	267.13	226.32
M247T356	(M+H)+	Asp-Leu	247.13	356.38
M233T386	(M+H)+	Val-Asp	233.11	386.32

M175T446	(M+H)+	L-Arginine	175.12	446.24
M203T258_2	(M+H)+	Ala-Leu	203.14	257.70
M213T411	$(M+CH_3CN+Na)+$	Triethanolamine	213.12	411.32
M281T224	(M+H)+	Val-Tyr	281.15	223.90
M218T411	(M+H)+	Ala-Gln	218.11	411.36
M290T443	(M+H)+	Asp-Arg	290.14	443.18
M223T262	(M+H)+	Phe-Gly	223.11	261.71
M310T292	$(M+CH_3CN+H)+$	Phe-Cys	310.13	292.27
M260T370	(M+H)+	Lys-Leu	260.20	370.13
M245T45	$(M+H-H_2O)+$	Phe-Pro	245.13	44.99
M276T406	(M+H)+	Thr-Arg	276.16	405.97
M199T67	$(M+H-H_2O)+$	Pro-Thr	199.11	66.91
M249T406	(M+H)+	Thr-Glu	249.11	405.96
M166T286	(M+H)+	L-Phenylalanine	166.09	285.62
M294T260	(M+H)+	Phe-Gln	294.14	259.75
M134T404	(M+H)+	L-Aspartate	134.04	404.29
M133T384	(M+H)+	L-Asparagine	133.06	383.88
M280T267	(M+H)+	Phe-Asn	280.13	266.81
M127T97	(M+H)+	Thymine	127.05	96.89
M285T417	(M+H)+	His-Glu	285.12	417.25
M303T52_2	$(M+H-H_2O)+$	20-Hydroxyarachidonic acid	303.23	51.92
M244T237	(M+H)+	Cytidine	244.09	236.62
M400T168	(M+H)+	L-Palmitoylcarnitine	400.34	168.12
M245T297_1	(M+H)+	Glu-Pro	245.11	296.51
M147T254_2	$(M+NH_4)+$	D-Pipecolinic acid	147.11	253.76
M237T233	(M+H)+	Ala-Phe	237.12	232.66
M303T280	(M+H)+	His-Phe	303.14	280.44
M220T272	(M+H)+	Pantothenate	220.12	271.72
M322T312_2	(M+H)+	Phe-Arg	322.19	312.11

M170T489	(M+H)+	1-Methylhistidine	170.09	489.23
M318T197	(M+H)+	Trp-Ile	318.18	196.85
M246T236	M+	2-Methylbutyroylcarnitine	246.17	235.99
M279T270	(M+H)+	Tyr-Pro	279.13	269.78
M295T144	(M+H)+	Tyr-Ile	295.16	143.72
M360T290	M+	Arg-Trp	360.19	289.83
M222T252	(M+H)+	N-Acetyl-D-glucosamine	222.10	251.80
M219T364	(M+H)+	Ser-Ile	219.13	363.98
M284T305	(M+H)+	Guanosine	284.10	305.11
M184T485	(M+H)+	Phosphorylcholine	184.07	484.51
M149T251	(M+H-H_2O)+	Phenyllactic acid	149.06	251.28
M407T90	(M+H)+	Lincomycin	407.22	89.53
M217T365	(M+H)+	N-alpha-Acetyl-L-arginine	217.13	365.07
M102T366	(M+H)+	1-Aminocyclopropanecarboxylic acid	102.05	365.89
M190T390	(M+CH_3COO+2H)+	L-Pyroglutamic acid	190.07	389.98
M249T220	(M+H)+	Val-Met	249.12	219.52
M232T503	(M+CH_3CN+H)+	N-(omega)-Hydroxyarginine	232.14	502.93
M247T281	(M+H)+	Pro-Met	247.11	281.22
M146T47	M+	2-Methylglutaric acid	146.06	46.59
M284T373	(M+H)+	His-Gln	284.13	372.87
M138T288_2	M+	Trigonelline	138.05	288.28
M215T315	(M+H)+	Pro-Val	215.14	315.32
M120T374	(M+H)+	L-Threonine	120.06	374.29
M289T511	(M+CH_3CN+H)+	Lys-Thr	289.18	511.31
M260T287	(M+H)+	Leu-Gln	260.16	287.04
M298T427	(M+CH_3CN+H)+	Thr-His	298.15	426.83
M269T158	(M+H-H_2O)+	His-Met	269.10	157.51
M148T381	(M+H)+	L-Glutamate	148.06	380.58
M201T405	(M+H-H_2O)+	5-L-Glutamyl-L-alanine	201.09	405.44

M189T384_2	(M+H)+	Leu-Gly	189.12	383.69
M146T499	M+	(3-Carboxypropyl)trimethylammonium cation	146.12	498.93
M213T361	(M+H)+	His-Gly	213.10	361.13
M276T439	$(M+H-H_2O)+$	Lys-Phe	276.16	438.93
M104T380	M+	Choline	104.11	380.49
M205T422	(M+H)+	Ala-Asp	205.08	422.38
M164T67	(M+H)+	Dimethylaminopurine	164.09	66.76
M261T459_2	$(M+H-H_2O)+$	Ile-Phe	261.15	459.43
M304T210	(M+H)+	Val-Trp	304.16	209.56
M296T36_2	$(M+NH_4)+$	alpha-Linolenic acid	296.26	35.74
M203T235	(M+H)+	Ile-Ala	203.14	234.69
M757T142	(M+Na)+	PC (16:0/16:0)	756.55	142.07
M311T370	(M+H)+	Tyr-Glu	311.12	369.71
M454T194	(M+H)+	1-Palmitoyl-2-hydroxy-sn-glycero-3-phosphoethanolamine	454.29	193.67
M166T388	(M+H)+	DL-Methionine sulfoxide	166.05	387.74
M284T470	(M+H)+	His-Lys	284.17	469.59
M297T193	(M+H)+	Phe-Met	297.12	193.04
M172T390	$(M+H-H_2O)+$	N-Acetyl-L-glutamate	172.06	389.93
M243T97	(M+H)+	Thymidine	243.10	96.76
M297T375	(M+H)+	Tyr-Asp	297.11	375.26
M306T360	(M+H)+	Met-Arg	306.16	360.50
M219T394	$(M+CH_3CN+Na)+$	Arecoline	219.11	393.87
M235T432	(M+H)+	Ser-Glu	235.09	432.25
M173T270	$(M+CH_3CN+H)+$	L-Isoleucine	173.13	269.58
M216T390	(M+H)+	sn-Glycerol 3-phosphoethanolamine	216.06	389.83
M221T422	(M+H)+	Ser-Asp	221.07	421.74
M329T206	(M+H)+	Phe-Tyr	329.15	205.55
M313T173	(M+H)+	Phe-Phe	313.15	172.79
M274T240_2	$(M+H-H_2O)+$	Cyclopentolate	274.17	239.55

M221T318_3	(M+H)+	Thr-Thr	221.11	318.13
M189T448	(M+H)+	N6,N6,N6-Trimethyl-L-lysine	189.16	447.71
M322T37	(M+H)+	Alpha-Linolenoyl ethanolamide	322.27	36.57
M271T420	(M+H)+	His-Asp	271.10	420.47
M217T246_2	(M+H)+	Val-Val	217.15	246.31
M263T258	(2M+H)+	L-Norleucine	263.19	258.13
M257T357	(M+H)+	His-Thr	257.12	356.96
M265T114	M+	Oxprenolol	265.16	114.23
M352T185	(M+H)+	Phe-Trp	352.16	184.85
M320T250	(M+CH_3CN+H)+	Pro-Tyr	320.16	250.50
M340T117	(M+H)+	N-Oleoylglycine	340.28	116.81
M231T424	(M+H)+	Pro-Asp	231.10	423.67
M278T383	(M+H)+	Met-Lys	278.15	382.93
M331T254_2	(2M+H)+	DL-Phenylalanine	331.16	254.36
M70T446	(M+H-2H_2O)+	Diethanolamine	70.06	446.00
M237T282	(M+H)+	Ser-Met	237.09	281.59
M482T186	(M+H)+	1-Stearoyl-2-hydroxy-sn-glycero-3-phosphoethanolamine	482.32	186.12
M247T34	(M+H-2H_2O)+	Oleic acid	247.24	34.37
M265T83_1	(M+H-H_2O)+	trans-Vaccenic acid	265.25	83.11
M338T374_2	(M+H)+	Tyr-Arg	338.18	373.51
M316T186	M+	Decanoyl-L-carnitine	316.25	186.00
M275T247	(M-H+2Na)+	Val-Leu	275.14	247.08
M281T346	(M+H)+	L-Aspartyl-L-phenylalanine	281.11	346.47
M303T521	(M+H)+	Arg-Lys	303.21	521.18
M203T481	(M+H)+	NG,NG-dimethyl-L-arginine(ADMA)	203.15	481.28
M246T359	(M+H)+	Val-Lys	246.18	358.65
M132T106	(M+H)+	L-Leucine	132.10	106.24
M312T456	(M+H)+	Arg-His	312.18	455.77
M184T46	(M+H)+	4-Pyridoxic acid	184.06	46.40

M267T418	$(M+H-H_2O)+$	Glu-His	267.11	418.04
M211T44	$(M+H-H_2O)+$	Ile-Pro	211.14	43.52
M100T277	$(M+H-H_2O)+$	5-Aminopentanoic acid	100.07	277.48
M342T280	(M+H)+	His-Trp	342.15	280.16
M245T158	(M+H)+	Uridine	245.08	158.36
M261T158_1	$(M+H-H_2O)+$	Ggamma-Glutamyl-L-methionine	261.08	158.35
M362T44	(M+H)+	Arachidonoylglycine	362.27	44.41
M139T289	(M+H)+	Urocanic acid	139.05	289.45
M150T124	(M+H)+	N6-Methyladenine	150.08	124.40
M209T200	$(M+H-H_2O)+$	His-Ala	209.10	200.26
M132T425	(M+H)+	Creatine	132.08	424.78
M112T252	(M+H)+	Cytosine	112.05	252.46
M428T165	(M+H)+	Stearoylcarnitine	428.37	164.57
M224T147	$(M+CH_3CN+H)+$	Dacarbazine	224.12	147.36
M211T244_1	(M+Na)+	Glycyl-L-leucine	211.11	243.72
M136T375	$(M+H-H_2O)+$	Dopamine	136.07	374.72
M187T378	(M+H)+	Ala-Pro	187.11	378.32
M243T60	(M+H)+	Lumichrome	243.09	59.82
M118T81	(M+H)+	L-Valine	118.08	81.06
M304T386	(M+H)+	Arg-Glu	304.16	386.21
M130T229	(M+H)+	Beta-Homoproline	130.08	229.25
M231T360_2	(M-H+2Na)+	Pro-Ala	231.07	359.58
M293T388	(M+H)+	His-His	293.13	388.08
M221T242	(M+H)+	Met-Ala	221.09	242.10
M204T501	(M+H)+	Gly-Lys	204.13	501.12
M333T269	(M+H)+	Trp-Gln	333.15	269.14
M150T209	(M+H)+	L-Methionine	150.06	209.44
M87T372	(M+H)+	4-Hydroxybutanoic acid lactone	87.04	372.42
M301T187	$(M+H-H_2O)+$	His-Tyr	301.13	186.70

M244T424	(M+H)+	Lys-Pro	244.16	424.04
M112T287	(M+H)+	Histamine	112.09	287.32
M550T183	M+	1-O-(cis-9-Octadecenyl)-2-O-acetyl-sn-glycero-3-phosphocholine	550.38	182.84
M247T411_1	(M+H)+	Gamma-L-Glutamyl-L-valine	247.13	411.09
M262T479	(M+H)+	Lys-Asp	262.14	479.06
M275T454	(M+CH_3CN+H)+	Lys-Ser	275.17	453.70
M303T439	(M+H)+	Arg-Gln	303.18	439.07
M208T200	(M+H-H_2O)+	6-Benzylaminopurine	208.10	200.19
M186T104	(M+H-2H_2O)+	N-Acetylmannosamine	186.07	103.93
M157T49	(M+H)+	3-Indoleacetonitrile	157.07	49.34
M114T48_2	(M+H)+	Epsilon-Caprolactam	114.09	47.52
M245T286	(M+H)+	Ile-Ile	245.18	285.96
M284T99	(M+K)+	Ile-Asn	284.10	98.71
M173T415_2	(M+H)+	Pro-Gly	173.09	414.67
M163T107	(M-H+2Na)+	2-Butoxyethanol	163.07	107.01
M189T407_2	(M+H)+	N6-Acetyl-L-lysine	189.12	406.87
M173T355_2	(M+H)+	Glycylproline	173.09	355.01
M195T223	(M+H-H_2O)+	Gly-His	195.09	222.90
M303T131	(M+H)+	Eicosapentaenoic acid	303.23	130.71
M158T77	(M+H-H_2O)+	Indoleacetic acid	158.06	77.19
M279T93	(M+H)+	cis-(6,9,12)-Linolenic acid	279.23	93.47
M239T303	(M+H)+	Tyr-Gly	239.10	302.75
M152T283	(M+H)+	2-Hydroxyadenine	152.05	282.90
M106T432	(M+H)+	L-Serine	106.05	431.93
M288T283	(M+H-H_2O)+	Arg-Met	288.15	282.78
M302T388	(M+CH_3CN+H)+	Ile-Glu	302.17	387.80
M265T185	(M+H)+	Phe-Val	265.15	184.80
M336T66	(M+H)+	Isopentenyladenosine	336.16	65.58
M258T138	(M+H)+	2'-O-methylcytidine	258.10	138.07

M587T90	(M+Na)+	Cer(d18:1/18:1(9Z))	586.52	89.61
M341T187	(M+H-H_2O)+	1-Stearoyl-rac-glycerol	341.30	186.71
M182T279	(M+H)+	L-Tyrosine	182.08	278.81
M156T292	(M+H)+	L-Histidine	156.07	292.37
M158T344	(M-H+2Na)+	Creatinine	158.03	343.55
M306T280	(M-H+2Na)+	Ser-Arg	306.12	280.44
M283T145	(M+H)+	2'-O-methylinosine	283.10	144.87
M302T270	(M+H)+	Pro-Trp	302.15	270.42
M303T224_2	(M+CH_3COO+2H)+	His-Ser	303.14	223.87
M270T211	(M+H-2H_2O)+	Capsaicin	270.18	211.49
M311T417	(M+H-H_2O)+	Tyr-Phe	311.13	417.24
M314T32	(M+CH_3CN+H)+	16-Hydroxypalmitic acid	314.28	32.14
M227T185	(M+K)+	L-NG-Monomethylarginine	227.09	185.44
M83T292	(M+H-H_2O)+	Glutaraldehyde	83.05	291.55
M113T111	(M+H)+	Uracil	113.03	111.21
M220T113	(M+H)+	trans-Zeatin	220.12	113.32
M232T529	(M+H)+	Gly-Arg	232.14	529.22
M191T314	(M+H)+	Thr-Ala	191.10	314.03
M337T549	M+	Arg-Tyr	337.17	549.10
M277T397	(M+H)+	gamma-L-Glutamyl-L-glutamic acid	277.10	396.78
M263T266	(M-H+2Na)+	Ile-Ser	263.10	266.34
M115T391	(M+H-H_2O)+	Ornithine	115.09	390.65
M248T420	(M+H)+	Thr-Lys	248.16	420.47
M330T152	M+	Eicosapentaenoic Acid ethyl ester	330.26	152.19
M188T223_1	(M+H-H_2O)+	DL-Indole-3-lactic acid	188.07	222.82
M74T251	(M+H)+	Methylguanidine	74.07	250.52
M114T400	(M+H-H_2O)+	N-Acetyl-L-alanine	114.05	400.37
M146T359	(M+H)+	4-Guanidinobutyric acid	146.09	359.00
M137T140	(M+H)+	Hypoxanthine	137.04	139.94

M229T111	(M+H)+	2'-Deoxyuridine	229.08	110.87
M316T80	$(M+NH_4)+$	Pristanic acid	316.32	80.22
M319T273_2	(M+H)+	Trp-Asn	319.14	272.97
M190T213	(M+H)+	Kynurenic acid	190.05	212.87
M122T162	(M+H)+	Phenylethylamine	122.09	162.28
M568T183	(M-H+2Na)+	1-Stearoyl-2-hydroxy-sn-glycero-3-phosphocholine	568.34	183.49
M169T290	(M+H)+	Pyridoxamine (PM)	169.10	289.96
M360T462	(M+K)+	Arg-Phe	360.14	462.42
M313T312	(M+H)+	Tyr-Met	313.13	312.11
M217T315	(M+H)+	Thr-Pro	217.12	315.27
M281T233	(M+H)+	Met-Met	281.10	232.90
M313T225	$(M+CH_3COO+2H)+$	2'-Deoxyinosine	313.12	224.95
M247T217	$(M+NH_4)+$	Pro-Asn	247.14	217.31
M153T78	(M+H)+	N1-Methyl-2-pyridone-5-carboxamide	153.06	78.30
M298T195	(M+H)+	2'-O-methylguanosine	298.11	194.93
M190T129	(M-H+2Na)+	Spermidine	190.12	129.36
M126T247	(M+H)+	Taurine	126.02	246.70
M310T268	(M+H)+	Tyr-Gln	310.15	268.10
M146T283	(M+H)+	4-acetamidobutanoate	146.08	282.89
M235T55_1	$(M+H-H_2O)+$	His-Pro	235.11	54.55
M263T113	(M+H)+	Ile-Met	263.15	112.77
M251T339	(M-H+2Na)+	Thr-Ser	251.06	338.62
M203T291_1	(M+H)+	Pro-Ser	203.10	290.68
M298T291_2	$(M+NH_4)+$	Phe-Asp	298.14	290.75
M242T155	M+	Pentadecanoic Acid	242.22	154.63
M400T256	$(M+CH_3CN+H)+$	Matairesinol	400.18	256.48
M391T196	(M+H)+	Trp-Trp	391.17	196.19
M277T318	$(M+H-H_2O)+$	gamma-L-Glutamyl-L-phenylalanine	277.13	318.09
M120T58	$(M+H-H_2O)+$	Tyramine	120.08	57.51

M232T243	$(M+H-H_2O)+$	Lys-Cys	232.12	242.84
M317T152	(M+Na)+	Ile-Tyr	317.15	151.51
M187T461	$(M+H-H_2O)+$	Gly-Glu	187.07	460.64
M251T196	(M+H)+	Thr-Met	251.11	196.18
M305T447	(M+H)+	N-Acetylaspartylglutamate (NAAG)	305.10	447.00
M628T198_1	$(M+H-H_2O)+$	1-Stearoyl-2-arachidonoyl-sn-glycerol	627.53	197.54
M186T159	$(M+H-H_2O)+$	Gly-Gln	186.09	159.13
M273T445	$(M+H-H_2O)+$	Argininosuccinic acid	273.12	444.92
M256T227	$(M+CH_3CN+H)+$	d-Dethiobiotin	256.16	226.92
M258T224	(M+H)+	3-methylcytidine	258.11	224.13
M263T74	$(M+H-H_2O)+$	Linoleic acid	263.24	73.58
M102T247	(M+H)+	Betaine aldehyde	102.09	247.34
M235T312	(M+H)+	Thr-Asp	235.09	311.95
M252T118	$(M+NH_4)+$	Glu-Ser	252.12	117.55
M192T243	(M+H)+	N-Acetyl-L-methionine	192.07	242.72
M377T211	(M+H)+	(-)-Riboflavin	377.14	210.95
M313T47	$(M+CH_3COO+2H)+$	Phe-Ser	313.14	47.03
M234T282	(M+H)+	Ser-Gln	234.11	282.13
M204T313	(M+H)+	Acetylcarnitine	204.12	313.36
M168T290	$(M+CH_3CN+Na)+$	(R)-3-Hydroxybutyric acid	168.06	290.02
M368T289	(M+H)+	Trp-Tyr	368.15	289.36
M548T254	(M-H+2Na)+	1-Eicosatrienoyl-sn-glycero-3-phosphoethanolamine	548.27	254.10
M146T218	$(M+H-2H_2O)+$	DL-O-tyrosine	146.06	218.41
M297T434	(M-H+2Na)+	Tyr-Ala	297.08	433.79
M231T105	(M+H)+	Leu-Val	231.17	104.79
M532T189_2	(M+Na)+	1-O-Octadecyl-sn-glyceryl-3-phosphorylcholine	532.37	188.63
M810T169	(M+Na)+	N-Docosanoyl-4-sphingenyl-1-O-phosphorylcholine	809.65	168.90
M258T171	(M+H)+	3'-O-methylcytidine	258.11	170.84
M180T286	M+	Theobromine	180.06	285.88

M142T227_3	(M+CH_3CN+H)+	4-Hexen-1-ol, (E)-	142.12	226.77
M272T313	(M+H-H_2O)+	Arg-Asp	272.13	313.43
M263T176	(M+H)+	.alpha.-L-Glu-L-Asp	263.08	175.75
M326T80	(M+H)+	N-Oleoylethanolamine	326.30	80.24
M219T321	(M+CH_3CN+H)+	N-Formylmethionine	219.08	321.49
M496T109_1	(M+H)+	1-Palmitoyl-sn-glycero-3-phosphocholine	496.34	109.35
M231T314_2	(M+H-2H_2O)+	Desipramine	231.16	314.01
M497T286	(2M+K)+	Propazine	497.17	285.95
M315T578	(2M+Na)+	L-Lysine	315.20	577.56
M152T158	M+	2-Methoxybenzoic acid	152.05	157.56
M344T353_2	(M-2H+3Na)+	Arg-Cys	344.08	352.96
M265T389	M+	Thiamine	265.11	388.56
M243T253	(M+K)+	Val-Ser	243.08	253.21
M153T109	(M+H)+	Xanthine	153.04	108.99
M297T225_1	(M+CH_3COO+2H)+	Phe-Ala	297.14	225.24
**ESI-**				
M141T332_2	(M-H_2O-H)-	2-Oxoadipic acid	141.02	332.35
M295T53	(M-H)-	9R,10S-EpOME	295.23	53.42
M255T46_2	(M-H)-	Palmitic acid	255.23	46.35
M293T50	(M-H)-	13-OxoODE	293.21	50.24
M179T259	(M-H)-	D-Fructose	179.06	259.01
M165T116_2	(M-H)-	DL-3-Phenyllactic acid	165.06	115.89
M131T130	(M-H)-	Hydroxyisocaproic acid	131.07	130.18
M331T41	(M-H)-	Adrenic Acid	331.26	41.18
M295T36	(M-H)-	9(S)-HODE	295.23	35.83
M351T103	(M-H)-	Prostaglandin H2	351.22	102.50
M329T42	(M-H)-	7Z, 10Z, 13Z, 16Z, 19Z-Docosapentaenoic acid	329.25	42.48
M105T314_2	(M-H)-	Glyceric acid	105.02	314.17
M103T410	(M-H)-	Malonic acid	103.00	409.83

M309T41	(M-H)-	2E-Eicosenoic acid	309.28	41.20
M607T43	(2M-H)-	Arachidonic Acid (peroxide free)	607.47	42.52
M172T214_2	(M-H)-	Acetyl-DL-Leucine	172.10	213.88
M133T409	(M-H)-	L-Malic acid	133.01	409.20
M181T297	$(M+CH_3COO)^-$	D-Threitol	181.07	296.73
M487T159_1	(2M-H)-	3,3',4,5'-Tetrahydroxy-trans-stilbene	487.13	158.88
M143T57_2	(M-H)-	Caprylic acid	143.11	56.78
M171T104	(M-H)-	Capric acid	171.14	103.67
M161T259	$(M-H_2O-H)^-$	D-Tagatose	161.05	259.00
M451T149	$(M+CH_3COO)^-$	Chenodeoxycholate	451.32	149.25
M175T380_2	(M-H)-	2-Isopropylmalic acid	175.06	380.34
M239T447	(M-H)-	L-Anserine	239.11	447.28
M190T219	(M-H)-	N-Acetyl-DL-methionine	190.05	219.20
M179T388_2	(M-H)-	myo-Inositol	179.06	387.83
M297T102_3	(M-H)-	Nname, cis-9,10-Epoxystearic acid	297.24	101.63
M204T175_1	(M-H)-	Indolelactic acid	204.07	175.31
M163T55	(M-H)-	Phenylpyruvate	163.04	55.43
M261T235	(M-H)-	L-phenylalanyl-L-proline	261.12	234.77
M206T200	(M-H)-	N-Acetyl-L-phenylalanine	206.08	199.60
M339T28	(M-H)-	Norethindrone Acetate	339.20	28.15
M383T256	M-	N-Acetyl-D-lactosamine	383.14	256.42
M153T55	(M-H)-	2,3-Dihydroxybenzoic acid	153.02	55.44
M181T167	(M-H)-	L-Iditol	181.07	166.83
M227T102_3	(M-H)-	Myristic acid	227.20	102.32
M173T360	(M-H)-	Suberic acid	173.08	360.25
M103T237	(M-H)-	D(-)-beta-hydroxy butyric acid	103.04	237.15
M167T116	(M-H)-	Homogentisic acid	167.03	116.03
M465T27	(M-H)-	Cholesteryl sulfate	465.30	26.53
M173T299_2	M-	Hexanoylglycine	173.10	299.32

M117T162	(M-H)-	2-Methyl-3-hydroxybutyric acid	117.06	161.57
M365T37	(M-H)-	Nervonic acid	365.34	37.14
M147T396	(M-H)-	(S)-2-Hydroxyglutarate	147.03	396.07
M158T220	(M-H)-	Acetyl-DL-Valine	158.08	220.23
M151T52	(M-H)-	2-Hydroxyphenylacetic acid	151.04	52.17
M129T476	(M-H)-	Citraconic acid	129.02	476.25
M321T102	$(M+CH_3COO)^-$	D-Mannitol 1-phosphate	321.17	101.65
M151T279	(M-H)-	Oxypurinol	151.03	278.64
M160T79	(M-H)-	Indole-3-carboxylic acid	160.04	79.07
M310T252	$(M+CH_3COO)^-$	Muramic acid	310.11	251.81
M209T297	$(M+CH_3COO)^-$	D-Lyxose	209.07	296.71
M174T132	M-	Shikimate	174.06	131.67
M128T210	(M-H)-	L-Pipecolic acid	128.07	209.86
M181T216	(M-H)-	Hydroxyphenyllactic acid	181.05	216.16
M281T143	(M-H)-	3'-O-Methylinosine	281.09	143.41
M116T281	(M-H)-	Acetylglycine	116.04	281.42
M129T227	$(M-H_2O-H)^-$	Dihydroxyfumarate	129.06	226.75
M301T44	(M-H)-	Eicosapentaenoic Acid	301.22	44.35
M187T346	(M-H)-	Azelaic acid	187.10	346.03
M151T103_2	$(M+CH_3COO)^-$	Glycerol	151.06	102.63
M151T240_2	(M-H)-	Xylitol	151.06	239.90
M171T435	(M-H)-	Glycerol 3-phosphate	171.01	435.31
M116T182	(M-H)-	Indole	116.05	182.30
M337T160	(M-H)-	Erucic acid	337.31	160.28
M133T146	(M-H)-	2,3-Dihydroxy-3-methylbutyric acid	133.05	146.20
M383T197	(M-H)-	Cholestenone	383.33	197.08
M358T284	$(M+NH_4-2H)^-$	Trehalose	358.14	284.25
M187T122	(M-H)-	3-Hydroxycapric acid	187.13	121.57
M175T313	$(M+CH_3COO)^-$	alpha-ketoisovaleric acid	175.06	313.14

M307T102	(M-H)-	11(Z),14(Z)-Eicosadienoic Acid	307.26	102.41
M561T194	(M-H)-	Protoporphyrin IX	561.25	193.77
M274T53_2	$(M+NH_4-2H)^-$	1-Methylpseudouridine	274.11	53.34
M463T249	(2M-H)-	Alantolactone	463.27	249.03
M174T396	(M-H)-	N-Acetyl-L-aspartic acid	174.04	396.04
M229T442	$(M+CH_3COO)^-$	Dihydroxyacetone phosphate	229.01	442.06
M180T126	(M-H)-	Acamprosate	180.03	125.67
M205T463	(M-H)-	Homocitrate	205.03	462.82
M177T107	(M-H)-	L-Gulonic gamma-lactone	177.04	106.62
M257T141	(M-H)-	Ribothymidine	257.08	141.14
M133T223	$(M-H_2O-H)^-$	Ribitol	133.05	223.20
M207T446	$(M+CH_3COO)^-$	Mevalonic acid	207.09	446.16
M152T345	(M+Na-2H)-	3-Guanidinopropanoate	152.04	345.01
M131T296	(M+CH3COO)-	Pyruvaldehyde	131.03	295.86
M171T345	(M-H)-	Sulfanilamide	171.03	344.92
M269T102_2	(M-H)-	Heptadecanoic acid	269.25	102.02
M257T162	(M-H)-	2'-O-Methyluridine	257.08	161.62
M283T164	(M-H)-	Stearic acid	283.26	164.33
M191T581	(M-H)-	Citrate	191.02	581.30
M161T375	(M-H)-	3-Hydorxy-3-methylglutaric acid	161.05	375.34
M197T380	$(M+CH_3COO)^-$	Fosfomycin	197.02	380.29
M107T52	(M-H)-	p-Cresol	107.05	52.18
M132T510	(M-H)-	D-Aspartic acid	132.03	510.42
M114T365	(M-H)-	Maleamic acid	114.02	364.74
M173T415	(M-H)-	N2-Acetyl-L-ornithine	173.09	414.72
M191T88	$(M-H_2O-H)^-$	Sedoheptulose	191.06	87.64
M212T53	(M-H)-	Indoxyl sulfate	212.00	53.40
M322T439	(M-H)-	Cytidine 5'-monophosphate (CMP)	322.04	438.57
M209T380	(M+CH3COO)-	D-Ribose	209.07	380.42

M104T446	(M-H)-	DL-Serine	104.04	445.80
M147T479	(M-H)-	Citramalic acid	147.03	478.74
M313T450	(M+Na-2H)-	Acetylvalerenolic acid	313.15	449.82
M243T239	(M-H)-	Pseudouridine	243.06	239.28
M89T357	(M-H)-	DL-lactate	89.02	356.67
M117T287	(M-H)-	Succinate	117.02	287.22
M154T319	$(M+NH_4-2H)^-$	4-Hydroxybenzoate	154.05	318.97
M125T144	(M-H)-	5-Amino-4-carbamoylimidazole (AICA)	125.05	144.13
M215T170	(M+K-2H)-	L-Galactono-1,4-lactone	215.09	170.17
M296T138	(M-H)-	3'-O-methylguanosine	296.10	137.97
M99T375	$(M-H_2O-H)^-$	3-Hydroxyisovaleric acid	99.04	375.26
M166T33	(M-H)-	Pyridoxal (Vitamin B6)	166.05	32.88
M233T30	(M-H)-	Confertifoline	233.15	30.21
M195T454	(M-H)-	Galactonic acid	195.05	453.73
M581T238	(M-H)-	Biliverdin	581.24	237.96
M138T226	(M-H)-	4-Nitrophenol	138.02	225.94
M426T468	(M-H)-	Adenosine 5'-diphosphate (ADP)	426.02	468.47
M375T283	(M-H)-	Riboflavin	375.12	283.49
M144T354	$(M+NH_4-2H)^-$	Dihydrothymine	144.08	354.07
M611T495	(M-H)-	Glutathione disulfide	611.14	495.02
M362T443	(M-H)-	Guanosine 5'-monophosphate (GMP)	362.05	443.37

m/z, mass-charge ratio; FH, fatty ham; LH, lean ham.
